# Twentieth century morality: The rise and fall of moral concepts from 1900 to 2007

**DOI:** 10.1371/journal.pone.0212267

**Published:** 2019-02-27

**Authors:** Melissa A. Wheeler, Melanie J. McGrath, Nick Haslam

**Affiliations:** 1 Department of Management and Marketing, Faculty of Business and Economics, University of Melbourne, Victoria, Australia; 2 Melbourne School of Psychological Sciences, Faculty of Medicine, Dentistry and Health Sciences, University of Melbourne, Victoria, Australia; The University of Memphis, UNITED STATES

## Abstract

Trends in the cultural salience of morality across the 20^th^ century in the Anglophone world, as reflected in changing use of moral language, were explored using the Google Books (English language) database. Relative frequencies of 304 moral terms, organized into six validated sets corresponding to general morality and the five moral domains proposed by moral foundations theory, were charted for the years 1900 to 2007. Each moral language set displayed unique, often nonlinear historical trajectories. Words conveying general morality (e.g., good, bad, moral, evil), and those representing Purity-based morality, implicating sanctity and contagion, declined steeply in frequency from 1900 to around 1980, when they rebounded sharply. Ingroup-based morality, emphasizing group loyalty, rose steadily over the 20^th^ century. Harm-based morality, focused on suffering and care, rose sharply after 1980. Authority-based morality, which emphasizes respect for hierarchy and tradition, rose to a peak around the social convulsions of the late 1960s. There were no consistent tendencies for moral language to become more individualist or less grounded in concern for social order and cohesion. These differing time series suggest that the changing moral landscape of the 20^th^ century can be divided into five distinct periods and illuminate the re-moralization and moral polarization of the last three decades.

## Introduction

Moral judgments and intuitions feature prominently in everyday life. They saturate the news, social media, and ordinary conversation and argument. Increasingly they also feature prominently in the academic literature. Morality has become a major interdisciplinary research focus [1], explored intensively within the fields of economics [2], philosophy [3], and evolutionary biology [4] among others.

Within the psychological study of morality, two key intellectual developments have been the ‘intuitionist turn’ [1] and the emergence of pluralist accounts. The former development reflects the growing recognition of the importance of emotion and intuition in moral judgment, in contrast to the rationalism of earlier approaches such as Kohlberg’s [5]. The latter development stemmed from cross-cultural research that broadened the scope of morality beyond individualistic considerations of harm and fairness [6] to include values associated with sociality and spirituality [7]. The idea that morality is not monolithic has had its most influential realization in the form of Moral Foundations Theory (MFT) [8, 9]. When first propounding MFT, Haidt and Joseph [10] aimed to categorize the automatic and intuitive emotional reactions that commonly occur in moral evaluation across cultures, and they identified five psychological systems (or foundations): Harm, Fairness, Ingroup, Authority, and Purity.

Each moral foundation has a distinct set of associated concerns, vices, and virtues. The Harm foundation includes concerns of cruelty, the suffering of others, and the virtues of compassion, caring, and kindness. Fairness covers issues of injustice, unfair treatment, reciprocity, equality, cheating, and individual rights. The Ingroup foundation is concerned with loyalty and obligations for group membership, self-sacrifice, and betrayal. Authority refers to social order, an obligation to conform to hierarchical relationships, and obedience and respect for authority and tradition. The Purity foundation is sensitive to contagion, both physical and spiritual, and encompasses concerns of sanctity, self-control, and the virtues of innocence and wholesomeness [1, 9]. Graham, Haidt, and Nosek [11] proposed that the foundations form two higher-order clusters of moral foundations. Harm and Fairness, the two *Individualizing* foundations, focus on the rights of the autonomous individual. Ingroup, Authority, and Purity, the three *Binding* foundations, have a collective focus on group cohesion.

Many studies have used the MFT framework to explore the ideological political divide between liberal and conservative moralities or moral values [6, 9, 11]. For example, Graham et al. [11] found that political liberals endorsed the Individualizing Harm and Fairness foundations, whereas conservatives endorsed all five foundations (including higher endorsement of the three Binding foundations when compared to liberals). To date, MFT has been applied to many different research questions and contexts. For example, numerous studies have explored the individual difference correlates of endorsement of the foundations [12], how endorsement varies as a function of relational context [13], or how foundation-specific moral judgments are communicated [14]. The breadth and quantity of this work shows MFT’s generativity as an account of the moral domain [15].

One largely unexplored dimension of the moral foundations is historical. Almost all previous research has examined use of the foundations at one point in time, and only one study has investigated temporal changes. Garten and colleagues [16] examined shifts in moral foundation language use in US political speeches from 1988 to 2012. Garten et al. investigated the moral foundations-related words that Democrats and Republicans used in the vicinity of the word ‘gay’. They found that Republicans were significantly more likely to use Purity-related words than were Democrats and showed a significant increase in Purity language in the period from 1996–2004 relative to the previous eight years. A broader and more fundamental question would be to ask whether and how the foundations have changed in their cultural salience over historical time.

Large scale, culture-level questions of this sort are ideally suited to ‘big data’ methodologies in moral psychology [17], such as explorations of vast linguistic corpora that have been dubbed examples of ‘culturomics’ [18]. A popular tool for such analyses is Google NGram Viewer, which allows users to gather word frequencies from the Google Books corpus of digitized books. These frequencies represent the proportion of any given input word within the corpus in any given year, thereby allowing rises and falls in relative frequency–an index of cultural salience or popularity–to be tracked over long periods of time. Psychological researchers have employed the tool to explore historical shifts from third person to first person pronouns [19–21], and changing concepts related to age stereotypes [22] and happiness [23]. Greenfield [24] demonstrated an ‘us’ to ‘me’ shift in word frequencies in English-language books published from 1800 to 2000 and revealed other patterned changes indicative of a movement from collectivist rural values to values that are more individualistic and urban. Zeng and Greenfield [25] replicated this shift in Chinese-language books, finding that words linked to materialism and individualism increased between 1970 and 2008, whereas words associated with collectivist values decreased.

Findings such as these appear to reveal important macro-level changes in cultural norms and values. However, none of them to date have directly examined changes in morality itself. The one exception is a pair of studies by Kesebir and Kesebir [26] that examined changes in moral virtues over time by recording the frequencies of moral excellence- and virtue-related words in the American corpus of Google Books using the NGram Viewer. Their first study documented a decrease in general virtue-related words (e.g., morality, conscience, character). Their second study examined fifty virtues of a more specific nature (e.g., honesty, kindness, trustworthiness), revealing a significant decline in 74% of those virtues over the course of the 20^th^ century. These studies reveal important historical trends in the cultural salience of some moral concepts but are limited in their attention to positive (virtue) concepts alone, their focus on specific terms rather than broader patterns linked to theoretical accounts of morality, and their emphasis on linear change trajectories (i.e., increases or decreases) rather than more complex, nonlinear patterns.

Building on past work, the present study investigated historical shifts in the cultural salience of multiple domains of morality, as revealed by changes in the relative frequency of large sets of moral terms within the Google NGram database of English language books. The five moral foundations and general morality were each represented by large, validated sets of individual words, and patterns of aggregate change within each set were examined for the years 1900 to 2007. The study’s aim was primarily descriptive and exploratory, seeking to characterize the potentially complex and nonlinear patterns of change in the moral domain across the 20^th^ century, patterns that have yet to be mapped. The analysis aimed to link these cultural trajectories tentatively to broader historical conditions, and to divide the 20^th^ century into periods based on distinctive configurations of particular moral domains.

Although the study was primarily exploratory, it was guided by three research questions. First, we asked whether general morality (i.e., words referring directly to good and bad, ethics and evil) had declined in salience over the course of the 20^th^ century consistent with the findings of Kesebir and Kesebir [26]. Second, we asked whether moral foundations associated with social cohesion and order (Binding foundations) tended to decline in relative frequency, whereas those associated with individual welfare and rights (Individualizing foundations, respectively) tended to increase. This expectation is consistent with past findings of rises in indicators of individualism and falls in indicators of collectivist values over the 20^th^ century [19, 20, 24, 25, 27], and also with the pronouncements of historians and social theorists. Prominent historian Eric Hobsbawm [28], for example, argued that “the cultural revolution of the latest twentieth century can thus best be understood as the triumph of the individual over society, or rather, the breaking of the threads which in the past had woven human beings into social textures”. Third, at the level of individual moral foundations, the study asked whether harm-based morality has become more salient since the rights revolutions of the 1960s [29], as Haslam [30] proposed in his work on ‘concept creep’. This work argues that the meanings of harm-related psychological concepts such as abuse, bullying, and trauma have broadened in response to rising cultural sensitivity to harm over the past half-century, a rise that would be expected to register in the growing salience of harm-based morality.

## Method

Six sets of terms (whole words or word stems) representing distinct forms of moral discourse were drawn from the Moral Foundations Dictionary, created by Graham et al. (2009) to be used with the Linguistic Inquiry and Word Count program (LIWC) [31]. Five dictionaries corresponded to the moral foundations (one for each foundation: Harm, Fairness, Ingroup, Authority, Purity) and one to “general morality” [11]. The moral foundations dictionaries divided terms into positive “virtue” terms and negative “vice” terms, each representing the distinctive content of valued and disvalued concepts within the respective foundations, designed to measure the different ways that people discuss their foundation-specific intuitions. Dictionary development [11] involved both an expansive and a contractive phase. In the expansive phase six researchers generated associations, synonyms, and antonyms for the core concepts for each of the foundations: harm and care, fairness and reciprocity, ingroup and loyalty, authority and respect, and purity and sanctity. Words that were only distantly related or had primary meanings without moral connotations (e.g., ‘just’) were deleted during the contractive phase. The dictionaries were validated by four raters scoring passages containing a subset of the words for contextual relevance.

Several terms from the moral foundations dictionaries (20 of 295; 6.8%) appeared in more than one dictionary. These were removed so that all moral foundations dictionaries contained only non-overlapping terms (mean dictionary size = 55 words; range 39 [Ingroup] to 80 [Purity]). The general morality dictionary contained a broader set of 41 morally saturated terms. Twelve of these terms also appeared in one of the moral foundations dictionaries, but these overlapping terms were retained in the general morality set because its temporal trajectory was not to be compared directly to those of the moral foundations. The final sample of 304 unique terms, organized in the six sets, is listed in S1 Table.

Relative frequencies in the Google Books database of each of the 304 terms for the years 1900 to 2007 were drawn from Google NGram Viewer. The default corpus, “English 2012”, was used, representing books in the English language published in any country. Information on the distribution of country of publication and on changes in that distribution across the study period is not available. Most books were drawn from university libraries and the database includes half a trillion English words. This database yields frequencies up to 2008, but that year’s data were found to show substantial discontinuities with preceding trends and were therefore excluded. Relative frequencies were calculated with ±3 year smoothing to reduce the jaggedness of the time series, as the goal of the analysis was to identify changes in frequency of words over extended periods rather than short-term fluctuations. NGram does not directly generate frequencies for word stems, which composed approximately half (51.0%) of the 304 terms. For each of these terms, NGram was used to select the three most frequently used whole words within the study period (based on mean relative frequency over the 108 years) and the relative frequencies of these three terms were aggregated as detailed below.

The relative frequency data generated by NGram were scaled prior to further analysis. The year in which each word achieved its highest relative frequency received a score of 100, and all other years were scaled so that their score represented a percentage of that peak value. In the case of terms that were word stems rather than whole words, the frequencies of their three highest-frequency whole word representatives were summed and these summed scores were then re-scaled by the same method. This process ensured that each word stem received a single series of scores scaled in the same way as the whole word terms. By this means, a dataset was constructed containing 304 terms in each of 108 years, where values represented each term’s relative frequency as a percentage of its highest elevation during that period.

The scaled terms in each set were then averaged for each year and their trajectories (time series) were plotted. These average values have a straightforward interpretation: if the value is 10 points higher in one year than another, then the average term in the set was 10% of the maximum relative frequency higher in the former than in the latter. Other methods of scaling terms (e.g., standardizing individual terms prior to averaging them) would not allow such ease of interpretation. The resulting averaged plots therefore represent broad and systematic tendencies, aggregated over large numbers of terms, for conceptually related forms of moral language to vary in cultural salience over historical time.

## Results

Prior to presenting our descriptive data analyses, we conducted preliminary checks on the internal consistency of the six sets of moral terms across the 108 years, and on the temporal coherence of their time series. Spearman Brown reliabilities based on random split-half correlations indicated excellent internal consistency for five of the sets (Authority = .93, harm = .90, Ingroup = .98, Purity = .99, General morality = .99). However, the Fairness set comprehensively lacked consistency, generating a negative split-half correlation of -.82. This finding does not imply that the Fairness set would necessarily lack consistency when assessing language use cross-sectionally, just that its words do not exhibit consistent patterns of historical change. Analysis of auto-correlations of each of the six time series demonstrated that their temporal variation was highly predictable rather than random. Auto-correlations at a four-year lag–the shortest lag outside the ±3 year smoothing band, which artifactually inflates auto-correlations at shorter lags–were lowest for Fairness (0.60) and otherwise ranged from 0.70 for Authority, to 0.81 for Harm and 0.90 for both Ingroup and General Morality. In sum, with the important exception of Fairness, the sets of moral terms demonstrated internal and temporal coherence.

Descriptive statistics, correlations with year, and intercorrelations among the six sets of moral terms over the 108 years are presented in Table 1 (all correlations are Pearson correlations with a critical value of *p* < .01 in view of the large number of correlations computed). The correlations with year reveal broad linear temporal trends in each set, and the intercorrelations show the degree to which the time series for different sets have similar or dissimilar overall trajectories. The standard deviations indicate that the Fairness, Authority, and Harm foundations had relatively low variability over time, whereas the Ingroup and Purity foundations and the General Morality dictionary had more substantial variability.

**Table 1:** Descriptive statistics and intercorrelations of the six moral dictionaries (1900–2007).

	Mean (SD)	Year	Harm	Fairness	Ingroup	Authority	Purity
Harm	66.33 (4.59)	-.06	-				
Fairness	70.51 (1.63)	.24	-.64*	-			
Ingroup	62.73 (7.24)	.97*	.12	.40*	-		
Authority	67.57 (3.24)	-.37*	.16	-.01	-.28*	-	
Purity	64.14 (8.71)	-.88*	.37*	-.53*	-.89*	.56*	-
General morality	66.67 (9.46)	-.92*	.37*	-.47*	-.93*	.45*	.98*

* *p* < .01

The six sets of moral terms had markedly different directions of change over the 20^th^ century. The Ingroup foundation demonstrated an overall rise, Harm and Fairness were relatively stable, whereas Authority and especially Purity and General Morality declined. Interestingly, the temporal intercorrelations among the moral foundations were not consistent with the typical cross-sectional pattern of associations among the Individualizing (Harm & Fairness) and Binding (Ingroup, Authority, and Purity) foundations [32]. Despite both being Individualizing foundations, Harm and Fairness were negatively associated (potentially indicating substitution of one for the other over time), and although Authority and Purity were positively associated with one another they were both negatively associated with Ingroup morality. Ingroup was positively associated with the Individualizing Fairness foundation, and Harm was positively associated with the Binding Purity foundation. Although their interpretation is complicated by the measurement deficiencies of the Fairness term set, these findings indicate that historical changes in the cultural salience of the moral foundations are not structured by more general changes in Binding and Individualizing morality. Once again, such findings do not necessarily call into question the coherence of the Binding and Individualizing groupings proposed by moral foundations theorists. These groupings may capture the covariation structure of individual differences in morality assessed at one point in time but not the structure of temporal changes in culture-level morality.

**Fig 1:**
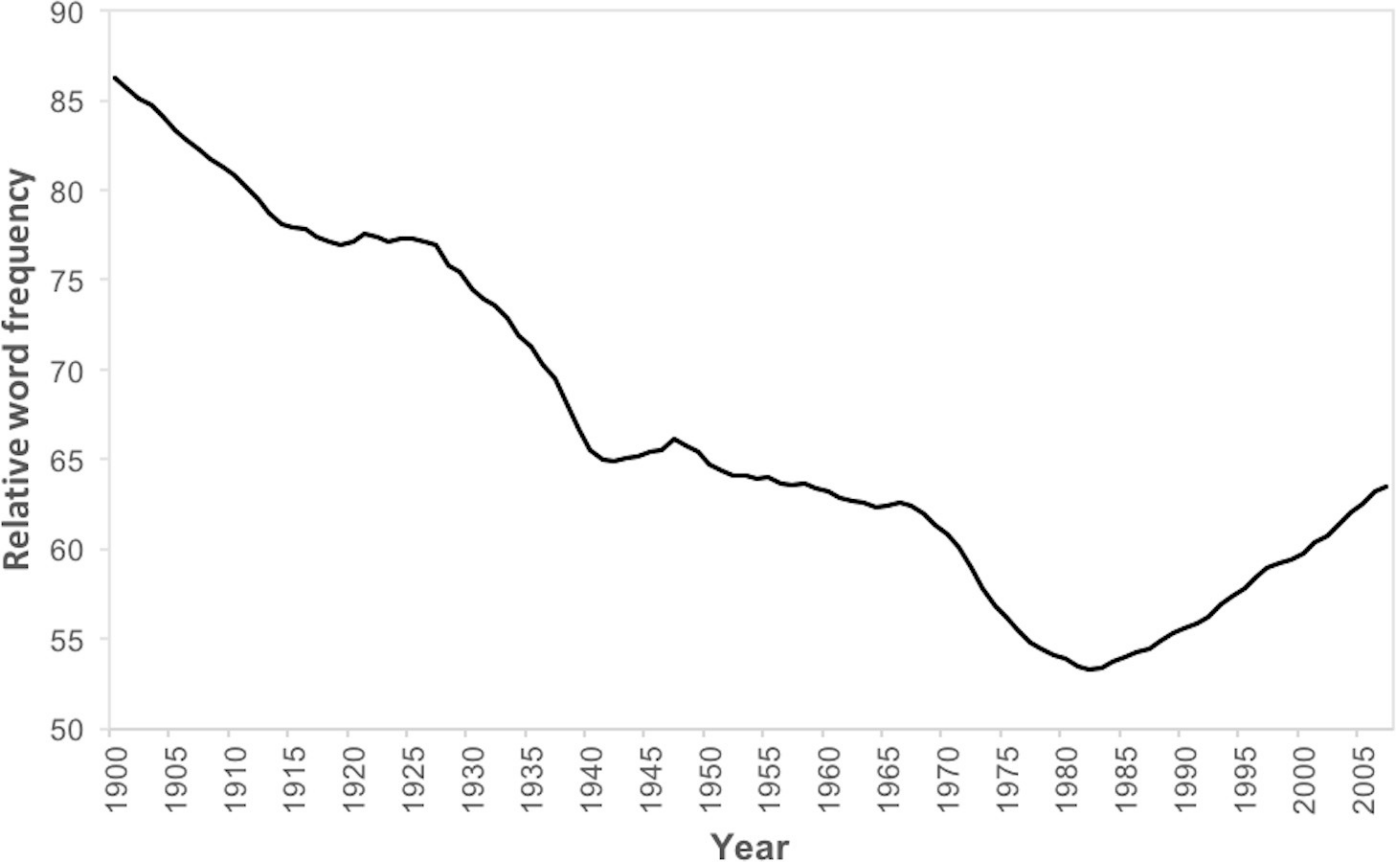
Time series of the general morality dictionary from 1900 to 2007.

To answer our first research question, we examined the trajectory of the General Morality set, which contained an assortment of morally-freighted terms (e.g., bad, character, ethic*, evil, good, immoral*, moral*, principle*, righteous*, value*, wrong*). Fig 1 reveals a steep decrease from 1900 to around 1980, consistent with the general decline in virtue language previously identified by Kesebir and Kesebir [26]. However, this decline is followed by an equally steep rise thereafter which was not identified in that earlier work. In effect, moral content in the Google Books database declined steadily throughout the 20^th^ century until an inflection point in around 1980. The subsequent rebound of moral language may point to the reinvigoration of social conservatism in the Anglophone world at around this time, led by such figures as Ronald Reagan and Margaret Thatcher, and manifest in the ongoing ‘culture wars’ and rising political polarization [8].

Our second research question concerned historical changes in broad Individualizing and Binding moral dimensions, represented by groupings of the five moral foundations. However, the lack of internal consistency of the Fairness foundation term set and the lack of consistent positive associations among the foundations within each grouping meant that this question could not be meaningfully addressed. In short, one of the two Individualizing foundations had serious measurement limitations and the Binding foundations did not form an internally coherent group. The second research question was therefore not addressed further and we proceeded to examine the time series for the five moral foundations independently.

**Fig 2:**
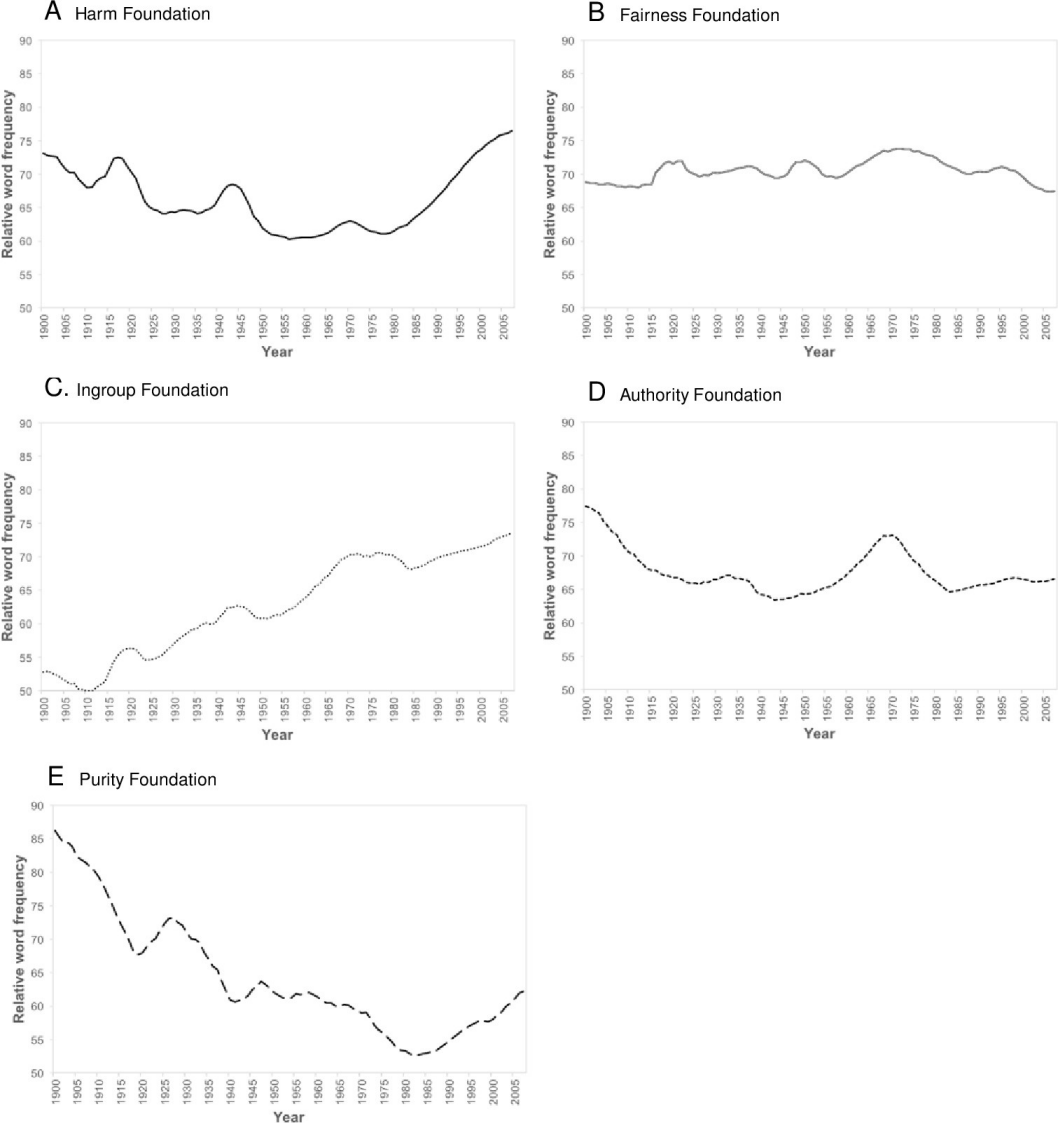
Time series of moral foundations indices from 1900 to 2007: (a) Harm, (b) Fairness, (c) Ingroup, (d) Authority, (e) Purity.

Time series for each moral foundation are presented in Fig 2. The five trajectories are very distinctive. Harm shows a gentle decline until about 1960, punctuated by noticeable short-term rises around the two World Wars, and then rises steeply from about 1980. This pattern is consistent with the expectation based on the third research question. Fairness, Harm’s fellow Individualizing foundation, has a relatively flat trajectory but begins to decline in the late 1970s, so that the two foundations trend in opposite directions in the last three decades of the study period. The problematic measurement properties of the Fairness term set make this discrepancy risky to interpret. Ingroup shows a relatively steady increase over the entire century. Authority declines until about 1950 then shows a striking rise to the late 1960s and an equally striking fall thereafter. The failure of Ingroup and Authority to rise in the vicinity of the World Wars as did Harm is perhaps surprising, as concern with loyalty and obedience might be expected to elevate during times of major conflict. Purity declines sharply from 1900 to about 1980 and then rises in a pattern that mirrors the trajectory of General Morality.

To formalize these descriptions of the shape of the six moral time series, we conducted a series of curve-fitting regression analyses. Three models were run for each series: the first included only a linear effect of time, the second added a quadratic effect, and the third added a cubic effect. Significance tests of these models are suspect due to the smoothing of the time series, but the explained variance added by each effect (see Table 2) offers a guide to interpreting the patterns described above. The General Morality and Purity series are both dominated by strong linear declines combined with smaller curvilinear (quadratic) effects representing their post-1970s rebounds. The Ingroup series shows a strong linear rise with no curvilinear trends. Harm and Fairness are dominated by curvilinear (quadratic) trends, the former concave and rising late in the 20^th^ century and the latter convex and falling in that period. Authority shows the most complicated curve, with a dominant cubic term to capture the circumscribed rise and fall of the foundation between 1950 and 1980. In sum, the moral time series are characterized by reliable curvilinear patterns, and linear, quadratic, and cubic terms collectively account for a very high proportion of their variance (*M* = 81.8%).

**Table 2:** Summary of curve-fitting analyses for the six moral dictionary time series.

	General morality	Harm	Fairness	Ingroup	Authority	Purity
Linear effect *R*^2^	.837	.003	.060	.943	.134	.765
Quadratic effect Δ*R*^2^	.102	.740	.398	.009	.154	.161
Cubic effect Δ*R*^2^	.027	.115	.104	.006	.344	.001
Total *R*^2^	.966	.859	.561	.959	.632	.928

To explore further the patterns observed for the five moral foundations, separate indices were computed for aggregates of their positive and negative terms (“virtues” and “vices”). Correlations between virtues and vices within foundations were strong for Purity (*r* = .99), Harm (*r* = .71), and Authority (*r* = .67), but relatively weak or negative for Fairness (*r* = .44, *p* < .001) and Ingroup (*r* = -.25, *p* < .01). Inspection of the 10 time series qualified several findings concerning the five foundations. First, the rise and fall of Authority on either side of the late 1960s is especially marked for its vice terms (e.g., defy*, disobe*, dissent*, rebel*). Second, Fairness vices (e.g., unequal*, unfair*, unjust*) display a pattern that is very similar to that of the Authority vices but was largely masked when the former were merged with Fairness virtues. They rise steeply from around 1955 to 1970 and then drop sharply. Finally, Ingroup virtues and vices reveal dissimilar time series. Ingroup vice terms (e.g., enem*, foreign*, immigra*, terroris*) are relatively static or in gentle decline from 1900 to the mid-1960s and then rise steeply. Ingroup virtue terms (e.g., communal, family, group, nation), in contrast, rise steeply from 1900 to around 1970 and then decline somewhat.

## Discussion

The temporal patterns revealed by our analysis are not consistent with a simple narrative of linear rises or falls in the cultural salience of morality through the 20^th^ century. Although the fundamental moral terms collected in the General Morality dictionary showed a steep decline, compatible with a broad reduction in the cultural salience of morality, that decline was not inexorable, reversing sharply from about 1980. According to the Google Books database, at least, the culture manifest in English language books has substantially de-moralized over the past century, but also re-moralized in the more recent past. This reversal of an otherwise striking decline in moral language, a nonlinear effect revealed as an aggregate pattern across 41 morally-saturated terms, is an important qualification to earlier findings that individual virtue-related moral terms have tended to decline in relative frequency [26].

A more complex narrative of an individualistic morality gradually replacing a morality based on social order and cohesion is also incomplete. Despite being opposed in theory [32], the Binding and Individualizing foundations did not form two coherent groupings. Neither of the Individualizing foundations (Harm and Fairness) displayed a rising linear trend between 1900 and 2007, contrary to previous findings on other indicators of individualism such as rising first-person pronoun use and materialist values [19, 20, 24, 25, 27], although Harm’s nonlinear trend showed a suggestive rise late in the 20^th^ century, consistent with prediction and Haslam’s [30] argument concerning ‘concept creep’. The Harm and Fairness time series were also negatively correlated, a finding inconsistent with Individualizing foundations being a coherent set, at least where patterns of historical change are concerned. However, the measurement deficiencies of the Fairness term set make it difficult to confidently interpret the negative correlation.

The three Binding foundations also lacked coherence as a group, and they had no consistent tendency to decline over the 20^th^ century. Purity demonstrated such a decline, reflecting a reduction–at least until around 1980 –in the salience of sanctity-based morality, but Ingroup morality showed a steady rise, and Authority displayed a complex nonlinear trajectory. Thus, our findings do not offer support for the view that Individualizing and Binding morality have shown a wholesale rise and fall, respectively. The most that could be claimed in that regard is that one form of Individualizing morality (Harm) and one form of Binding morality (Purity) display this pattern, and the former rise was only evident from about 1980 onward. Thus, a key finding of our study is that the five moral foundations have unique, often nonlinear trajectories that cannot be aggregated into broader groupings. Each form of morality captured by a foundation appears to have its own irreducible historical patterning.

Although our finding that the Individualizing and Binding foundations lacked empirical coherence as groupings might be taken as evidence against the validity of the groupings themselves, we would caution against that inference. Although Individualizing and Binding may not accurately capture the covariation structure of historical changes in morality at the cultural level, they may still validly describe the structure of covariation in morality assessed at one point in time, and/or assessed at the level of individual differences. There is ample evidence that Individualizing and Binding foundations cohere empirically in other research contexts, and are related to additional factors such as political orientation in divergent ways [11, 15]. Consequently we do not see our findings as posing a fundamental challenge to moral foundations theory.

The complex patterns revealed by the five moral foundations can be very tentatively integrated into five historical periods. The first period, covering 1900 to WWI and corresponding to the final stages of the Belle Époque, is one of liberalization, illustrated by reductions in the Purity and Authority foundations. The second, interwar period, which includes two convulsive conflicts and the Great Depression, interrupts this liberalizing process. The Binding foundations, particularly Purity and Ingroup, rise and then fall during this period, suggesting a preoccupation with protecting the cohesion and sanctity of group-based identities.

The third period, from around the end of WWII to around 1968, can be interpreted as a gathering crisis of authority. Three foundations rise in parallel: Authority and Fairness (both especially for vice terms), and Ingroup (especially virtue terms). These linked increases signify a growing cultural preoccupation with disobedience, rebellion, injustice, and inequality, coupled with an increasing focus on loyalty to groups. The fourth period, from around 1968 to around 1980, represents a second liberalization. The Binding foundations (especially Authority and Purity) decline steeply and Harm begins a steady rise that points to a growing concern with suffering, care, and protection of the vulnerable.

The fifth period, from around 1980 to the end of the study period in 2007, involves a relatively sudden shift in the salience of moral concepts. The inflection point corresponds roughly to the beginning of more than a decade of uninterrupted conservative rule in the Anglophone heartlands of the USA and the UK. Moral content increasingly saturates the database and the Binding foundations–especially Purity but also Ingroup and Authority–reverse their previous decline. The Individualizing foundations, led by Harm, continue their increase, so that both individualist and social order and cohesion-based moralities rise in parallel, suggesting a broader re-moralization. This positively correlated increase of normally antagonistic moralities of the political left and right may point to increasing moral polarization and conflict [8].

Historical speculation of this sort can only be put forward with great care. Caution is required when drawing inferences regarding cultural trends from Google Ngram data. Although the corpus drawn from Google Books is the largest currently available there are several limitations in its sampling. First, the corpus reflects what has been published, not necessarily what is widely popular in the general public, and it is not specific to a single national ‘culture’. Second, some researchers have suggested that the Google Ngram English corpus has included progressively more scientific literature throughout the 20^th^ century [33]. This does not necessarily undermine the capacity of the data presented in the present study to illuminate broad cultural trends, and the fact that some forms of moral language increased over the course of the 20^th^ century arguably conflicts with the expectation that moral language would decline with the increased inclusion of relatively neutral scientific reports. Third, because the dataset represents books published in English during a century in which major Anglophone countries rose and fell in their relative cultural influence (e.g., the rise of the USA relative to the UK), it is possible that some of the historical trends observed in our study are confounded by national differences. Fourth and finally, some research has suggested there is a time lag of up to a decade between exogenous events and their effects in literature [34]. Future research could seek to replicate our findings with other corpora that potentially have different temporal relationships to exogenous variables, such as those that aggregate new media reports rather than books.

Although Moral Foundations and the accompany Moral Foundations dictionary is a widely used and understood taxonomy of moral priorities, there are other approaches to categorization in the moral domain. Further research using dictionaries based on alternative moral schema such as Janoff-Bulman and Carnes’ Model of Moral Motives [35] might reinforce the historical patterns suggested by our data or illuminate other trajectories of moral language.

## Conclusions

The present study adds to an emerging body of quantitative research on historical changes in human culture. It extends previous work with its thorough and systematic attention to the multiple dimensions of morality. The dynamic changes in the salience of morality through the 20^th^ century that it finds are complex, resisting simple linear narratives of uninterrupted rises or falls. There does appear to have been a progressive reduction in the cultural salience of morality in general since the beginning of the last century, but there has also been a vigorous rebound since the early 1980s. At a more fine-grained level, different moral foundations have markedly distinct trajectories, which correlate with major societal conflicts and developments. Understanding historical variations in moral judgments and values may help to illuminate social challenges in the present and those yet to arise.

## Supporting information

S1 Table Words and word stems employed in each moral foundation and general morality dictionary. (DOCX)
