# Supplementary material for: Twentieth century morality: The rise and fall of moral concepts from 1900 to 2007
Source: PLoS One. 2019 Feb 27;14(2):e0212267. doi: 10.1371/journal.pone.0212267 (PMC6392263; doi:10.1371/journal.pone.0212267)
Supplement: S1 Table — (DOCX) [file pone.0212267.s001.docx]

|  | **Harm** | **Fairness** | **Ingroup** | **Authority** | **Purity** | **General Morality** |
| --- | --- | --- | --- | --- | --- | --- |
| **Virtues** | amity  benefit*  care  caring  compassion*  defen*  empath*  guard*  peace*  protect*  safe*  secur*  shelter  shield  sympathy* | balance*  constant  egalitar*  equable  equal*  equity  equivalent  evenness  fair  fairly  fairmind*  fairness  fairplay  fair-*  homologous  honest*  impartial*  justice  justifi*  justness  reasonable  reciproc*  rights  tolerant  unbias*  unprejudice* | ally  cadre  cliqu*  cohort collectiv*  communal  commune*  communis*  communit*  comrad*  devot*  familial  families  family  fellow*  group  guild  homeland*  insider  joint  member  nation*  patriot*  solidarity  together  unison  unite* | abide  allegian*  authorit*  bourgeoisie  caste*  class  command  complian*  comply control  defer  defere*  duti*  duty  father*  hierarch*  honor*  law  lawful*  leader*  legal*  mother  mothering  motherl*  mothers  obey*  obedien*  order*  permission  permit  position  rank*  respect  respected  respectful*  respects  revere*  serve  status*  submi*  supremacy  tradition*  venerat* | abstemiousness  abstention  abstinen*  austerity  celiba*  chast*  church*  clean*  decen*  holiness  holy  immaculate  innocent  integrity  limpid  maiden  modesty  piety  pious  pristine  purity  pure*  refined  sacred*  saint*  steril*  unadulterated  upright*  virgin  virginal  virginity  virgins  virtuous  wholesome*  wicked*  wretched* | blameless  canon  character  commendable  correct  decen*  doctrine  ethic*  exemplary  good  goodness  honest*  ideal*  integrity  laudable  lawful*  legal*  lesson  moral*  noble  piety  pious  praiseworthy principle*  proper  righteous*  upright  upstanding  value*  wholesome*  worth* |
| **Vices** | abuse*  annihilate*  attack*  brutal*  cruel*  crush*  damag*  destroy  detriment*  endanger*  fight*  harm*  hurt*  impair  kill  killed  killer*  killing  kills  ravage  stomp  spurn  suffer*  violen*  war  warl*  warring  wars wound* | bias*  bigot*  discriminat*  dishonest  disproportion*  dissociate  exclud*  exclusion  favoritism  inequitable  injust*  preference  prejud*  segregat*  unequal* unfair*  unjust*  unscrupulous | deceiv*  enem*  foreign*  immigra*  imposter  individual*  jilt*  miscreant  renegade  sequester  spy  terroris* | agitat*  alienate  defector  defian*  defy*  denounce  disobe*  disrespect*  dissent*  dissident  illegal*  insubordinat*  insurgent  lawless*  mutinous  nonconformist  obstruct oppose  protest  rebel*  refuse  remonstrate  riot*  sediti*  subver*  unfaithful | adulter*  blemish  contagio*  debase*  debauche*  defile*  deprav*  desecrat*  dirt*  disease*  disgust*  exploitat*  filth*  gross  impiety  impious  indecen*  intemperate  lax  lewd*  obscen*  pervert  profan*  profligate  promiscu*  prostitut*  repuls*  sick*  sin  sinful*  sinned  sinner*  sinning  sins  slut*  stain*  taint*  tarnish*  tramp  trashy  unchaste  unclean*  wanton  whore | bad  evil  immoral  indecen*  offend* offensive* transgress*  wicked*  wretched* wrong* |
